# Catalpol: An Iridoid Glycoside With Potential in Combating Cancer Development and Progression—A Comprehensive Review

**DOI:** 10.1002/ptr.70057

**Published:** 2025-09-23

**Authors:** Lucas Fornari Laurindo, Victória Dogani Rodrigues, Elen Landgraf Guiguer, Lívia Fornari Laurindo, Debora Aparecida Pires de Campos Zuccari, Claudia Rucco Penteado Detregiachi, Adriano Cressoni Araújo, Jéssica da Silva Camarinha Oliveira, Durvanei Augusto Maria, Jefferson Aparecido Dias, Rose Eli Grassi Rici, Caroline Barbalho Lamas, Rosa Direito, Sandra Maria Barbalho

**Affiliations:** ^1^ Department of Biochemistry and Pharmacology School of Medicine, Universidade de Marília (UNIMAR) Marília São Paulo Brazil; ^2^ Department of Biochemistry and Pharmacology School of Medicine, Faculdade de Medicina de Marília (FAMEMA) Marília São Paulo Brazil; ^3^ Postgraduate Program in Structural and Functional Interactions in Rehabilitation, School of Medicine, Universidade de Marília (UNIMAR) Marília São Paulo Brazil; ^4^ Department of Biochemistry and Nutrition School of Food and Technology of Marília (FATEC) Marília São Paulo Brazil; ^5^ Department of Molecular Biology School of Medicine, Faculdade de Medicina de São José do Rio Preto (FAMERP) São José do Rio Preto São Paulo Brazil; ^6^ Development and Innovation Laboratory Butantan Institute São Paulo São Paulo Brazil; ^7^ Graduate Program in Anatomy of Domestic and Wild Animals College of Veterinary Medicine and Animal Science, Universidade de São Paulo (USP) São Paulo São Paulo Brazil; ^8^ Department of Gerontology School of Gerontology, Universidade Federal de São Carlos (UFSCar) São Carlos São Paulo Brazil; ^9^ Laboratory of Systems Integration Pharmacology, Clinical and Regulatory Science Research Institute for Medicines, Universidade de Lisboa (iMed.ULisboa) Lisboa Portugal; ^10^ Charity Hospital, Universidade de Marília (UNIMAR) Marília São Paulo Brazil

**Keywords:** cancer, catalpol, inflammation, metastasis, oxidative stress, phytotherapy

## Abstract

Catalpol, a natural iridoid glycoside known for its anti‐proliferative effects, has been proposed as an anticancer compound. Catalpol targets critical processes involved in cancer cell progression, like malignant proliferation, apoptosis, and metastasis. Additionally, catalpol presents potent anti‐inflammatory and antioxidant properties crucial for cancer prevention and intervention. Due to the absence of clinical trials, this review investigates twelve studies, encompassing in vitro and animal trials from reputable databases, such as PubMed, with no time restrictions. Therefore, we covered evidence from catalpol's effects against several types of cancer, including breast, liver, colorectal, lung, gastric, bladder, and ovarian cancer, as well as osteosarcoma, and assessed various outcomes related to cell viability, apoptosis, migration, and modulation of molecular mechanisms by catalpol. Notably, catalpol induced cancer cell death via induction of mitochondrial apoptosis pathways, regulation of the expression of specific microRNAs, modulation of Sirt1, Kras, RACK1, PARP, PI3K/Akt, Bcl‐2, and STAT3/JAK2/Src signaling pathways, and inactivation of NF‐kB and Smad 2/3 signaling pathways. Furthermore, catalpol limits cancer metastasis due to modulation of critical metalloproteinases associated with cancer migration. Catalpol also synergizes with chemotherapeutic and adjuvant agents to induce cancer control, including regorafenib in liver cancer and chloroquine in gastric cancer, promoting increased anticancer action via upregulated cancer cell apoptosis, decreased proliferation, and inhibited angiogenesis via PI3K/p‐Akt/mTOR/NF‐κB, VEGF/VEGFR2, and Bax signaling pathways modulation. Catalpol derivatives also gained attention. Pyrazole‐, imidazole‐, and hydrolyzed‐based catalpol derivatives increase cancer cell apoptosis and death and decrease tumor angiogenesis through similar pathways. This review seeks to provide understanding of catalpol's anticancer effects, its mechanisms of action, and its potential as a therapeutic anticancer agent while advocating for future research conductance.

## Graphical abstract

Catalpol's potential in inhibiting cancer development and progression through various pathways.

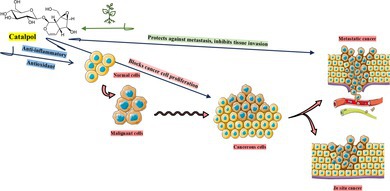

## 1 Introduction

Cancer remains one of the leading causes of death worldwide (Bray et al. 2024). The disease is characterized by uncontrolled cell proliferation and decreased cell death, negatively affecting an individual's well‐being and profoundly causing social, economic, and psychological implications (Peng et al. 2022; Więckiewicz et al. 2024). These abnormal cells often grow beyond their usual boundaries, invade surrounding tissues, and spread to other tissues and organs (Gerstberger et al. 2023). Cancer's pathophysiology involves genetic mutations and epigenetic alterations, which disrupt normal cellular processes regulating cell division, differentiation, and apoptosis (Chaudhry et al. 2022; Yu et al. 2024). Cancer‐related growth, spread, metabolite production, tissue disruption, and resource use may cause pain, organ failure, and other cancer‐related syndromes like cancer cachexia (Brown et al. 2023). It is well known that widespread metastasis is the leading cause of death from cancer (Seyfried and Huysentruyt 2013).

While advancements in cancer therapies have significantly improved patient outcomes, the challenges of curing cancer due to cancer heterogeneity and overall adaptability, limitations like drug resistance, side effects, variability across different cancer types, and, principally, the high costs of chemotherapy limit the efficacy of ongoing treatments. As a result, there is a need for continuing and future research for novel therapeutic agents that may overcome resistance, adverse effects, and the variability of cancer types (Barbalho et al. 2024; Laurindo, de Lima, et al. 2024; Laurindo, Pomini, et al. 2024; Laurindo, Sosin, et al. 2024). In this scenario, phytochemicals are a promising area of interest. Phytochemicals are bioactive, naturally occurring molecules derived from many species of plants. The predominant classes are terpenoids, phytosterols, phenolic constituents, polyphenols, alkaloids, fibers, saponins, and carotenoids (Rabizadeh et al. 2022; Rodrigues et al. 2024; Thakur et al. 2020). These bioactive compounds show promise in various human ailments without serious side effects, including cancer (Laurindo, de Maio, et al. 2023). Developing phytochemical‐based anticancer therapies involves the extraction, separation, purification, and testing of these molecules against different cancer cell lines, and translational research is of utmost importance (Laurindo, Barbalho, et al. 2023; Majrashi et al. 2023).

**FIGURE 1:**
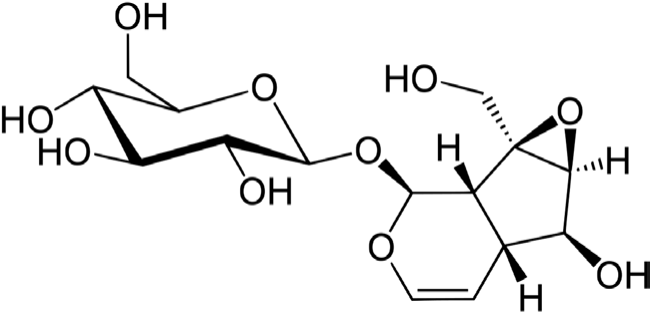
Molecular structure of catalpol.

Catalpol (Figure 1), an iridoid glycoside, is a phytochemical derived primarily from *Rehmannia glutinosa* (Huang, Gong, et al. 2022). However, the compound is distributed in various plant families, such as *Plantaginaceae*, *Lamiaceae*, and *Bignoniaceae* (Damtoft 1994; Kroll‐Møller et al. 2017; Lino von Poser et al. 2000). Iridoid glycosides are terpene‐derived bioactive compounds that possess a structure related to iridodial (Lohaus 2022). Typically, iridoids have been recognized as defense chemicals of plants against herbivores due to their bitter taste and anti‐growth inhibitory activities against insects and pathogens (Yamane et al. 2010). Because of its polar functional groups, catalpol is highly hydrophilic and soluble in water and methanol. Its glycosidic bonds make it unstable in acidic environments and susceptible to hydrolysis. Catalpol synthesis includes the mevalonic acid (MVA) or 2‐c‐methyl‐d‐erythritol‐4‐phosphate (MEP) pathways (Zhang, Dai, et al. 2023). Besides the typical role of defending plants against different insults, preclinical observations have made catalpol a potential intervention in health and disease, especially in targeting cancer due to its anti‐growth effects. Catalpol also has anti‐inflammatory (Liu, Liu, et al. 2023) and energy‐modulation properties (Zheng et al. 2024), neuroprotective (Yang et al. 2020), antidiabetic (Fu et al. 2023), immunomodulation effects (Wu et al. 2022), and hepatoprotective activity (Zhang, Ran, et al. 2024). Catalpol impacts many signaling pathways involved in key cellular processes, such as nuclear factor erythroid 2‐related factor 2 (Nrf2) (You et al. 2021), phosphatidylinositol 3‐kinase (PI3K) and protein kinase B (Akt) (Wang et al. 2021), mammalian target of rapamycin (mTOR) (Gao et al. 2023), adenosine monophosphate‐activated protein kinase (AMPK) (Laurindo et al. 2025; Zhang et al. 2021), nuclear factor kappa B (NF‐kB) and nucleotide‐binding and oligomerization domain‐like receptor family pyrin domain‐containing 3 (NLRP3) (Liang et al. 2023), vascular endothelial growth factor (VEGF)/vascular endothelial growth factor receptor 2 (VEGFR2) (El‐Hanboshy et al. 2021), sirtuin 1 (Sirt1) (Jiang et al. 2022), and others.

Previously published reviews have partially addressed catalpol's anticancer properties. Jiang et al. (2021) studied the effects of many phytochemicals derived from traditional Chinese medicine against breast cancer. However, their findings on catalpol were weak since several other bioactive constituents were evaluated, and they focused solely on breast cancer. On the other hand, Bhattamisra et al. (Bhattamisra et al. 2019) studied the biological effects of catalpol against multiple health and disease conditions, including cancer. However, their review was limited due to their limited inclusion of studies on catalpol targeting cancer. Xue et al. (Xue et al. 2019) reviewed the ethnopharmacology, phytochemistry, and pharmacology of iridoid glycosides from the genus *Veronica*; catalpol is one compound. However, their analysis focused not on catalpol and cancer but on aucubin and other catalpol derivatives on various health conditions like inflammatory, oxidative, infective, neurodegenerative, and hepatic diseases. More recently, Hassan et al. (2025) discussed the multifaceted effects of catalpol against various diseases, including diabetes, cardiovascular diseases, respiratory disorders, liver diseases, neurological outcomes, and cancer. However, their analysis of catalpol's effects on cancer is minimal as they just mention the anti‐proliferative potential of catalpol against cancer, lacking sufficient analysis and thorough exploration of this bioactive compound's effectiveness against malignancies and its molecular and pharmacological targets. This present manuscript aims to comprehensively review the effects of catalpol on cancer development and progression based on recent studies. This review is the first to focus solely on catalpol's impacts across different cancer lineages, highlighting its potential in combating cancer development and progression. We included all relevant studies, and, fortunately, all studies sought for retrieval were successfully retrieved. Our review encompasses many cancer lines derived from breast, liver, colorectal, gastric, ovarian, lung, bladder cancers, and osteosarcoma. By synthesizing findings from preclinical studies, we seek to highlight the novel insights and therapeutic potential of catalpol, fill existing literature gaps, and propose future research directions.

## 2 Catalpol: Unveiling Its Biosynthesis, Physicochemical Properties, and Pharmacokinetics

The content of catalpol differs between species, extraction methods, and plant parts. Previous research has indicated that fresh *R. glutinosa* contains 3%–4% catalpol, its dry roots 1.79%–2.28%, and significantly less, around 0.27% after steaming. Besides, the leaves of this plant are also rich in catalpol, with the latter containing around 3.81–24.51 mg/g of catalpol (Bai et al. 2019). *R. glutinosa* is an essential medicinal plant in Asia and is widely used as a herbal medicine, especially its roots. *R. glutinosa* roots present various diameters, lengths, weights, and catalpol differences between individuals immediately after harvest. Huang et al. found that catalpol content in *R. glutinosa* tended to increase as root diameter increased. In their experiments, they reported that catalpol content decreased with drying. Regarding root diameter, the average catalpol content for each group of root diameter was 0.5% for 1.0 to < 1.5 cm, 2.2% for 1.5 to < 2.5 cm, and 2.3% for 2.5 to ≤ 3.5 cm. Regarding the drying process, those researchers affirmed that the catalpol content was better preserved within the sliced roots that underwent air‐drying for five months compared to whole roots naturally dried for nine months (Huang, Ando, et al. 2022).

### 2.1 Biosynthesis of Catalpol: Pathways and Regulatory Mechanisms

Although catalpol presents many health benefits and modulates several signaling pathways, its biosynthesis remains obscure (Zhou et al. 2023). Generally, catalpol is synthesized via the MVA or MEP pathways. The initial synthesis process involves the head‐to‐tail condensation of isopentenyl pyrophosphate to dimethylallyl pyrophosphate (DMAPP) in a plastid. This step leads to geranyl diphosphate (GPP). Then, in the cytoplasm, the plastid is transformed into geraniol by catalytic cytochrome P450 reductase (CPR) and geraniol 10‐hydroxylase (G10H), generating 10‐hydroxy geraniol in the process. Geraniol is then converted into 10‐oxo geranial. At this point, 10‐oxo geranial is transformed into 8‐epi‐iridodial and then oxidized to 8‐*epi*‐iridotrial. The first process is catalyzed by iridoid synthase in an NADH/NADPH‐dependent manner. This intermediate underwent glycosylation and oxidation, producing epideoxyloganic acid. Epideoxyloganic acid is then hydroxylated at C‐8 and dehydrated, hydroxylated at C‐6 and C‐10, and decarboxylated into reactions that generate aubucin. Aubucin passes through an epoxidation between C‐7 and C‐8, generating catalpol. Natural catalpol derivatives can also be found within nature. Comparative analysis has demonstrated that *R. glutinosa* possesses more than 200 genes of 13 enzymes potentially involved in catalpol biosynthesis. The downstream process of catalpol formation also involves 22 genes for dehydratases, 10 for epoxidases, 19 for hydroxylases, and 30 for decarboxylases (Li, Zhai, et al. 2024; Zhang et al. 2019). However, the results are intricate since other additional studies have indicated that radial striation (RS) and non‐radial striation (nRS) from four *R. glutinosa* cultivars transcriptome sequencing were found to be differentially expressed in all RS versus nRS comparisons, 362 unigenes. Of these, 143 were upregulated and encoded other enzymes of the catalpol biosynthetic pathway, including geranyl diphosphate synthase (RgGPPS), phenylalanine ammonia‐lyase (RgPAL), and geraniol 8‐hydroxylase (RgG10H) (Zhi et al. 2018). Due to the diverse results from different sources, there is no consensus on a significant regulatory enzyme for catalpol biosynthesis.

### 2.2 Physicochemical Properties of Catalpol: Structural Characteristics and Stability

Catalpol is an iridoid glycoside with a polar structure, which indicates catalpol's uneven distribution of electrical charges and affects its interactions with other substances. Soluble in water and less stable in an acidic environment than alkaline, catalpol is unstable under higher temperatures, degrading rapidly at 100°C (2–6 h). Previous studies have also indicated that catalpol presents excellent stability in neutral solutions and sensitization to acids and alkalis. Besides being degraded under lower pH conditions, acid hydrolysis is also a result. Regarding the necessary activation energy for catalpol degradation, catalpol behaves differently than other glycosides, and the required energy under different pH values is all below that of acid‐catalyzed hydrolysis of general glycosides, which is about 138–142 kJ/mol (Bai et al. 2019; Wei and Wen 2014). According to PubChem (2004) by the National Library of Medicine from the United States (US) government, catalpol (PubChem ID 91520) is an organic molecular entity. It possesses a role as a metabolite, performing its roles in metabolic processes. Catalpol has a molecular formula of C_15_H_22_O_10_, a molecular weight of 362.33 g/mol, and an exact mass of 362.12129689 Da. It has a six‐hydrogen bond donor count and a ten‐hydrogen bond acceptor count. With a topological polar surface area of 162 Å^2^, catalpol presents a heavy atom count of 25, a complexity score of 542, an eleven‐defined atom stereocenter count, and no undefined atom stereocenter count. With an XLogP3‐AA of −3.2, the catalpol molecular structure is canonicalized. According to PubChem (2004) and the European Chemicals Agency (ECHA 2024), catalpol (EC/List no. 219‐324‐0) was reported as an irritant compound that causes skin irritation, severe eye irritation, and respiratory irritation. However, the physio‐pathological pathways are still not fully understood.

### 2.3 Pharmacokinetics of Catalpol: Evaluating the Phytochemical's Absorption, Distribution, Metabolism, and Toxicity

Accurate and efficient methods of catalpol detection are crucial for the correct research and development of its health effects, and understanding the underlying pharmacokinetic characteristics of catalpol is essential for the development of catalpol‐based drugs. Currently, the main techniques of catalpol detection are based on liquid chromatography, layer chromatography, micellar electrokinetic capillary chromatography, and chromatography‐tandem mass spectrometry. Due to its high polarity, catalpol presents limited ultraviolet (UV) absorption, which causes difficulty in the detection of UV high‐performance liquid chromatography (HPLC‐UV). Therefore, micellar electrokinetic capillary chromatography (MECC) mass spectrometry (MECC‐MS) is the method of choice regarding sensitivity and specificity for catalpol detection in plants and formulations. Previous studies have shown that catalpol is rapidly absorbed by the intestine after oral administration. However, its bioavailability is affected by the intestinal microbiota composition that converts catalpol (Tao et al. 2016, Zhang, Dai, et al. 2023). Its distribution throughout the bloodstream permits penetration through the blood–brain barrier (Wang et al. 2012; Xue et al. 2015). After metabolization by multiple reactions, including hydrogenation and deglycosylation, catalpol metabolites can be found in rat plasma and eliminated in rat urine and faeces (Tao et al. 2016; Xiang et al. 2021). Still, for chronic kidney disease and diabetes, catalpol's absorption appears to be accelerated, and catalpol's elimination appears to be slowed, suggesting elimination via diuresis (Feng et al. 2013; Zhao et al. 2015). Previous articles have indicated values for catalpol's pharmacokinetics in mice (Zhang, Dai, et al. 2023). Catalpol presents half‐life absorption rates from 0.53 ± 0.30 h in healthy rats and half‐life from 11.60 ± 4.20 h in diabetic rats after oral administration to 0.68 ± 0.24 h after intravenous administration in healthy rats. In addition, the compound achieves maximum concentration in diabetic rats from 37.41 ± 13.01 mg/L after oral administration, time to maximum concentration from 0.25 ± 0.10 h after intraperitoneal administration in healthy rats, and volume of distribution from 3.60 ± 1.40 L/kg after oral administration in healthy rats to 0.40 ± 0.20 L/kg after intraperitoneal administration in healthy rats. Regarding clearance, results indicate 4.90 ± 2.50 L/h/kg in healthy rats and 0.0002 ± 0.00 L/h/kg in chronic kidney disease rats after oral administration. Regarding bioavailability, results indicate values from 71.62% ± 10.28% in healthy rats after intraperitoneal administration to 66.70% in healthy rats after oral administration. Studies on catalpol's pharmacokinetics in humans are not available. Catalpol's 50% lethal dose was determined in the literature after intraperitoneal administration in Kunming mice as 206.5 mg/kg (He et al. 2021, Zhang, Dai, et al. 2023).

### 2.4 Pharmacodynamics of Catalpol: Mechanisms of Action and Therapeutic Potential

This subsection briefly describes catalpol's mechanisms of action against oxidative stress, inflammation, and other properties related to general health, going beyond specific cancer targeting by catalpol to fully demonstrate this iridoid glycoside's pharmacological potential. Although antioxidant and anti‐inflammatory effects, for example, are related to cancer prevention and intervention, within the following sections, all relevant studies on catalpol, specifically targeting cancer, will be appropriately assessed as the primary interest of the present manuscript. Catalpol fights inflammation through various mechanisms. It has been described that catalpol modulates the metabolism and inflammatory injury during myocardial injury via sirtuin 5 (Sirt5)‐mediated signaling, decreasing levels of different pro‐inflammatory cytokines like tumor necrosis factor‐alpha (TNF‐α), interleukin (IL)‐1β, and IL‐6 (Zheng et al. 2024). Against neuroinflammation, catalpol exerts beneficial effects through NF‐κB/NLRP3 modulation, inhibiting pro‐inflammatory cytokines like IL‐6, TNF‐α, and IL‐1β and modulating key inflammatory signaling molecules such as NLRP3, NF‐κB, caspase‐1, and apoptosis‐associated speck‐like protein containing a CARD (ASC) (She et al. 2024). During renal damage, catalpol ameliorates inflammation via inhibition of the toll‐like receptor 4 (TLR4)/myeloid differentiation primary response 88 (MyD88) signaling and uric acid reabsorption inhibition (Chen et al. 2024). Other signaling pathways modulated by catalpol to counteract inflammation are microRNA‐124‐3p (miR‐124‐3p)/deoxyribonucleic acid (DNA) methyltransferase three beta (DNMT3β)/tumor necrosis factor receptor‐associated factor six (TRAF6) (Zhang, Feng, et al. 2024), high mobility group box 1 (HMGB1)/TLR4/NF‐κB signaling pathway via the microRNA‐410‐3p (miR‐410‐3p) (Feng et al. 2023), and Nrf2/heme oxygenase‐1 (HO‐1) signaling (Zhang and Qiang 2023). Against oxidative stress, catalpol alleviates oxidative stress injury within the neurons via activating the Kelch‐like ECH‐associated protein one (Keap1)‐Nrf2/antioxidant response element (ARE) signaling pathway (Xiang et al. 2024), prevents atherosclerosis, protecting endothelial cells from hyperhomocysteinemia‐related damage through modulating reactive oxygen species (ROS)/NF‐κB signaling (Wu et al. 2024), and diminishes oxidative liver damage via regulating the Sirt1/hypoxia‐inducible factor 1α (HIF‐1α) pathway (Nie et al. 2024). Catalpol also exerts protective effects on blood vessel endothelial cells in trauma models through HIF‐1α/VEGF modulation (Ni et al. 2024). Other antioxidant mechanisms are related to Nrf2/HO‐1 activation (Lang et al. 2024), PI3K/Akt/Nrf2/HO‐1 upregulation (Wu, Wang, et al. 2021), and peroxisome proliferator‐activated receptor gamma (PPAR‐γ) activation (Jiang and Zhang 2020). Metabolically, catalpol inhibits aerobic glycolysis in the liver, modulating the Ephrin type‐A receptor 2 (EphA2)/focal adhesion kinase (FAK)/proto‐oncogene tyrosine‐protein kinase (Src) signaling pathway, which conserves liver structure and functionality (Zhang, Ran, et al. 2024). Catalpol also exerts hepatoprotective effects by downregulating the Janus kinase (JAK)/signal transducer and activator of the transcription (STAT) signaling pathway (Ji et al. 2024). Additionally, catalpol ameliorates diabetes and lipid dysfunctions by inhibiting microRNA‐101‐3p (miR‐101‐3p) to upregulate FOS‐related antigen 2 (FOSL2) (Xu et al. 2024). Catalpol also promotes antidiabetic effects via the AMPK signaling pathway (Li, Chen, et al. 2024). Against cardiovascular diseases, catalpol mainly modulates antioxidant pathways such as Nrf2/HO‐1 (Ge et al. 2022) and inhibits oxidative stress, myocardial apoptosis, and autophagy via modulation of the Akt–mTOR pathway (Liu, Xie, et al. 2023). Against neurological and neurodegenerative diseases, catalpol promotes neurogenesis and angiogenesis via the stromal cell‐derived factor 1 alpha (SDF‐1α)/C‐X‐C motif chemokine receptor 4 (CXCR4) pathway during ischemic alterations (Zhang, Wang, et al. 2024) and modulates ferroptosis in Alzheimer's disease through heat shock protein family A (Hsp70) member 5 (HSPA5)/glutathione peroxidase 4 (GPX4) axis intervention (Tian et al. 2024). Catalpol also induces osteogenic differentiation through the estrogen receptor alpha (ER‐α)/PI3K/Akt signaling pathway (Hu et al. 2024).

## 3 Catalpol in Cancer Prevention and Intervention

The present section delves into the recent evidence supporting catalpol's potential to influence vital cancer‐related processes. Catalpol affects cancer cell proliferation, cancerous apoptosis, and tumor metastasis, and can be considered a potential anticancer bioactive compound. With this present review, we aimed to highlight its mechanisms of action, assess its therapeutic potential, and discuss its implications for future cancer treatment strategies.

### 3.1 Literature Search Methodology: Paving the Way for Literature Exploration

A systematic and critical literature search was conducted across reputable databases, including PubMed, Scopus, Web of Science, Embase, and Google Scholar. The rationale behind the conduct of this comprehensive and systematic review delves into the fact that iridoid glycosides, bioactive compounds, often possess anticancer effects and, specifically, no published systematic reviews addressed catalpol's full anticancer potential based on the compound's demonstrated impacts and mechanisms of action, besides the growing amount of evidence indexed every year. Keywords were employed to pave the way for literature exploration. They included “catalpol,” “cancer cell lines,” “animal models,” “cancer,” “breast cancer,” “oral cancer,” “gastric cancer,” “lung cancer,” and “signaling pathways” alongside terms related to biological processes such as “apoptosis,” “cell proliferation,” “metastasis,” “PI3K,” “Nrf2,” “mTOR,” and “NF‐kB.” Given the lack of clinical trials on catalpol, the inclusion criteria comprised in vitro and in vivo studies focusing on catalpol's anticancer effects. The included studies must have examined the impact of catalpol on various cancer cell lines and animal models and covered diverse types of cancer. Cell viability, apoptosis, migration, treatment options and durations, administration routes, and modulation of molecular pathways relevant to cancer progression were outcomes of utmost interest. Exclusion criteria encompassed reviews, meta‐analyses, poster presentations, editorials, communications, letter to the editor, non‐experimental papers, and studies that did not involve catalpol as an intervention or were not based on cancer cell lines or relevant animal models. No time restrictions were applied to the literature search to emphasize a broader range of included studies. However, studies lacking robust experimental designs or transparent reporting of results were excluded from the final analysis. Two experienced researchers (L.F.L. and S.M.B.) who had conducted systematic reviews and meta‐analyses were responsible for data extraction from the included articles. Data extraction was performed using a standardized, systematic review method to capture essential information on experimental models fully, catalpol treatment details (concentration and duration), outcomes, and limitations. The PICO—Population, Intervention, Comparison, and Outcome—alongside limitations analysis, were imposed for data extraction. The included studies' design, sample size, and clarity of result reporting were assessed to evaluate the quality of the articles based on their experimental design, following the Preferred Reporting Items for Systematic Reviews and Meta‐Analyses (PRISMA)‐established guidelines for scientific rigor (Page et al. 2021). A qualitative data synthesis was conducted to summarize the effects of catalpol on cancer cell lines and animal models, identify expected outcomes, and discuss limitations. The findings were organized into different tables to highlight the anticancer effects of catalpol, mechanisms of action, and potential as a therapeutic agent. This review also aims to pinpoint gaps in the current research, suggest future directions for other research endeavors such as translational research, and promote catalpol's possible clinical applications in cancer prevention and intervention.

### 3.2 Literature Search Report: Results of Literature Search Following PRISMA Guidelines

**FIGURE 2:**
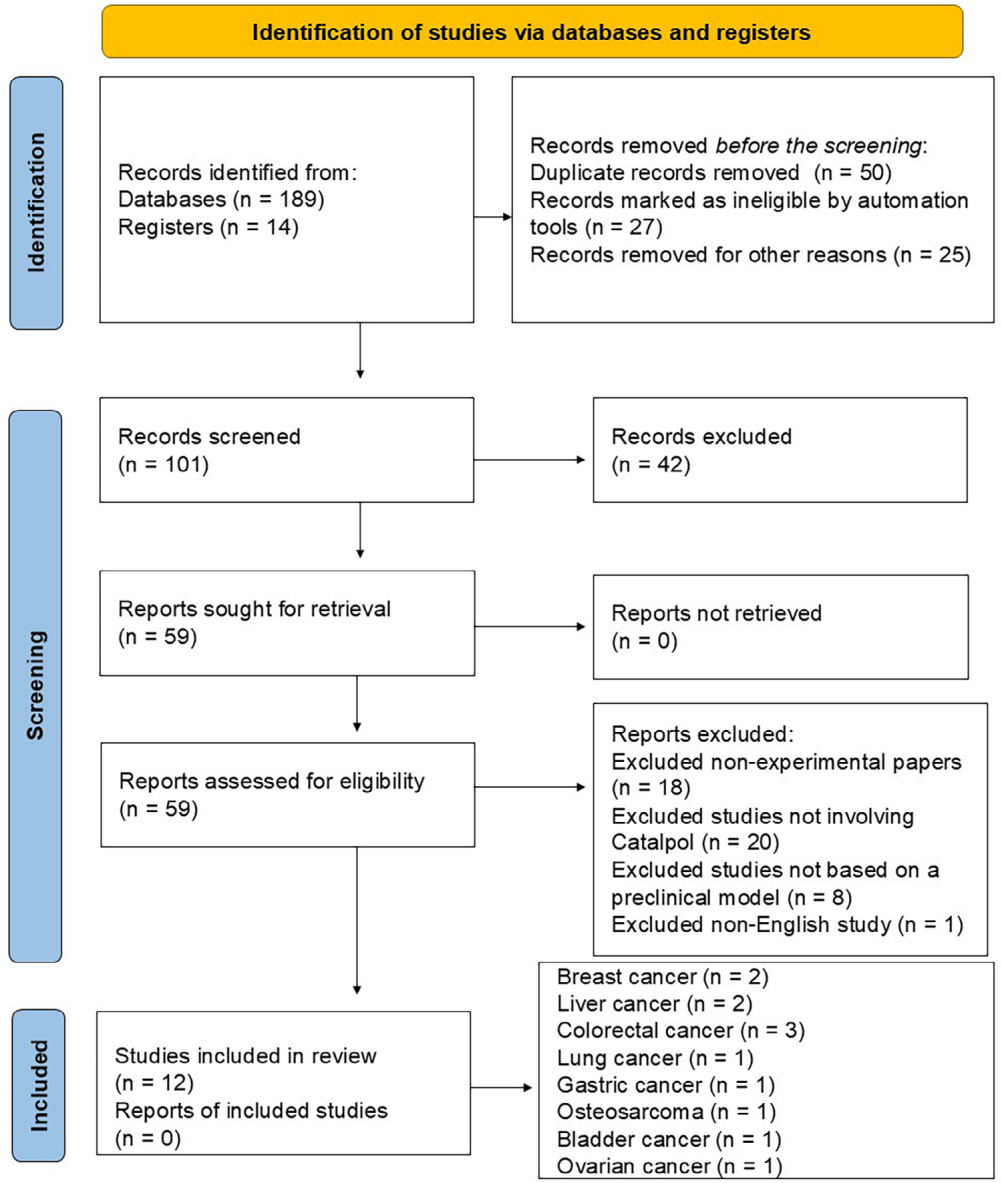
Flow diagram of the study selection process following PRISMA guidelines.

Records were identified from databases (*n* = 189) and registers (*n* = 14). This initial search resulted in a total of 203 records. Before the screening process, several records were removed: 50 duplicate records were eliminated using Covidence software, 27 were marked as ineligible by automation tools by PRISMA2020 software, and 25 were removed for other reasons based on their fragile methodology or incomplete results sharing. Consequently, 101 records remained for screening. During the screening process, 42 records were excluded based on the predefined criteria of being an experimental paper strictly due to the absence of clinical trials explicitly focusing on catalpol targeting cancer. The remaining 59 records were then sought for retrieval. Fortunately, all reports could successfully be retrieved, leaving the same 59 to be assessed for eligibility. Upon assessing these 59 reports for eligibility, further exclusions were made. Eighteen non‐experimental papers were excluded, 20 studies not involving catalpol were excluded, eight not based on a preclinical model were excluded, and one was excluded for not being in English. This resulted in 12 studies being included in the review, encompassing various types of cancer, such as breast cancer (*n* = 2), liver cancer (*n* = 2), colorectal cancer (*n* = 3), lung cancer (*n* = 1), gastric cancer (*n* = 1), osteosarcoma (*n* = 1), bladder cancer (*n* = 1), and ovarian cancer (*n* = 1). Figure 2 presents the study selection process flow diagram, following the PRISMA guidelines.

### 3.3 Preclinical In Vitro and In Vivo Anticancer Studies of Catalpol: Mechanisms, Efficacy, and Potential Clinical Implications

**FIGURE 3:**
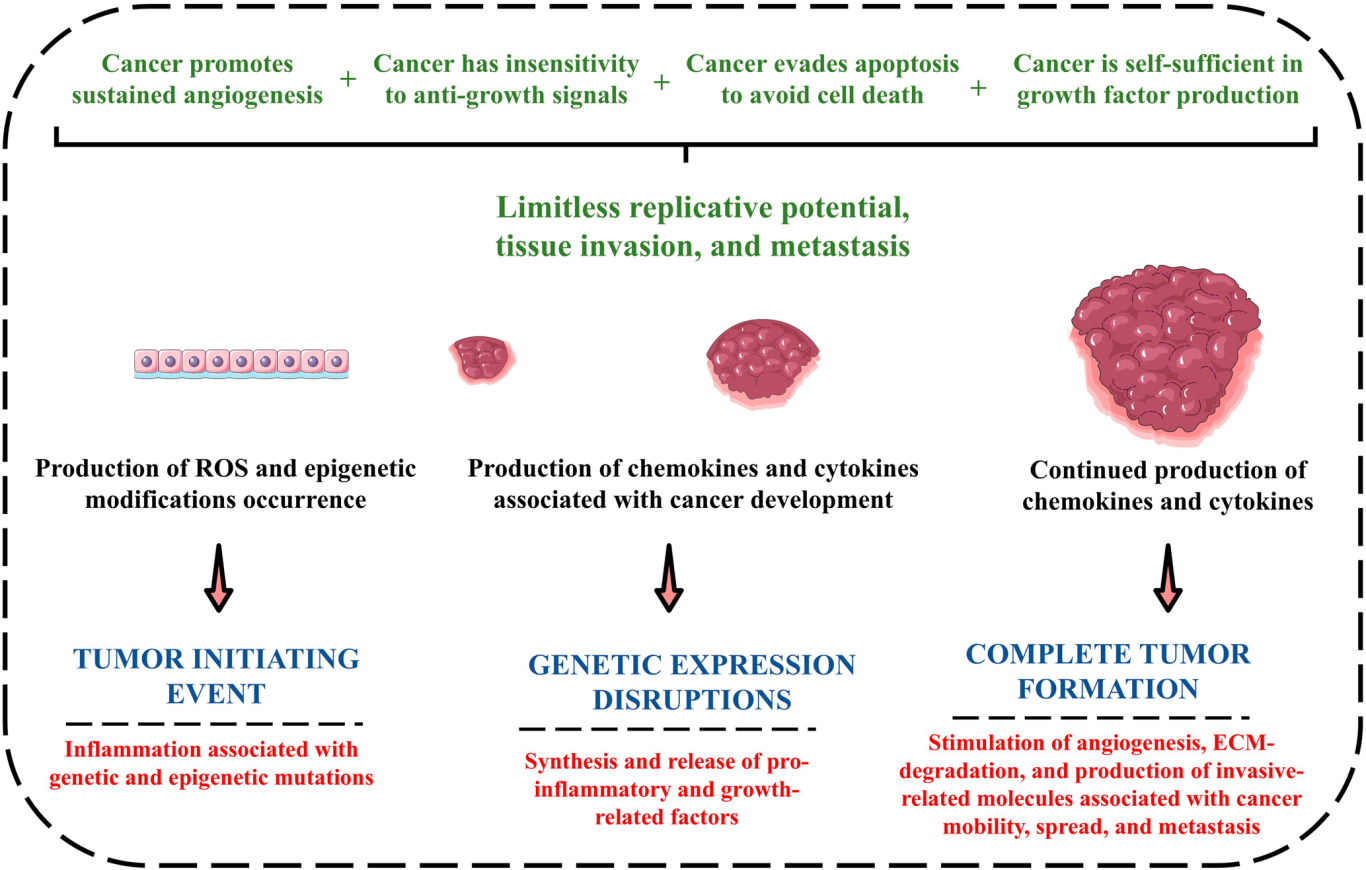
Mechanisms of cancer formation and metastasis. ECM, extracellular matrix; ROS, reactive oxygen species.

This comprehensive review elucidates catalpol's mechanisms of action in cancer prevention and intervention, highlighting its multifaceted mechanisms of action across various cancer cells and animal models. The gathered evidence underscores catalpol's potential as a therapeutic agent against different types of cancer, yet emphasizes the necessity for further validation, particularly in clinical settings using translational research and well‐designed clinical trials. Tables 1 and 2 summarize the effects of catalpol on various cancer cell lineages and animal models in detail, including the specific interventions, outcomes, limitations, and possible implications following PRISMA guidelines and the PICO framework. The most significant anticancer effects of catalpol are mainly modulating inflammation, angiogenesis, and apoptosis, alongside blocking the cancer cell cycle. To facilitate the understanding of the biological processes included, such as cancer apoptosis, proliferation, metastasis, and possible targets for catalpol interventions, Figure 3 illustrates the mechanisms of cancer formation and spread.

**TABLE 1:** In vitro anticancer effects and underlying mechanisms of action of catalpol.

Cell line/s	IC_50_/EC_50_/Conc. and duration	Effects demonstrated	Mechanisms of action	References
Breast cancer				
MCF‐7	0.25, 0.5, and 1 mM, 24, 48, and 72 h	┴ Cell proliferation and viability; induced apoptosis, loss of mitochondrial membrane potential, and enhancement of ROS generation; ↑ the level of cytoplasmic cytochrome c and activity of caspase‐3	Induction of the mitochondria apoptosis pathway, regulation of protein PTMs	(Liu et al. 2022)
MCF‐7	25, 50, and 100 μg/mL, 24, 48, and 72 h	↓ Cell proliferation, ↑ apoptosis	↑ Expression of miR‐146a, ↓ MMP‐16 activity	(Liu et al. 2015)
Liver cancer				
HCC (HCCLM3, Huh7)	50 μM (IC_50_), 48 h	┴ Cell proliferation, invasion, and migration; ↓ expression levels of vimentin and N‐cadherin; ↑ expression levels of E‐cadherin and miR‐140‐5p	Regulation of the expression of miR‐140‐5p	(Wu, Li, et al. 2021)
HCC (HCCLM3, Huh7)	50 μM (IC_50_), 48 h	┴ Cell viability and colony growth; ↓ invasion and migration; ↑ apoptosis, and proportion of cells at G0/G1 cell cycle phase	Modulation of the miR‐22‐3p/MTA3 axis	(Zhao et al. 2019)
Colorectal cancer				
CRC (FHC, HCT116, HT29, SW620, SW480)	10, 20, 30, 40, and 50 μM, 24 h	↓ Cell viability; ┴ autophagy; promoted apoptosis	Regulation of the expression of Sirt1 by inducing miR‐34a	(Qiao et al. 2020)
HCT116	25, 50, and 100 μg/mL, 24, 48, and 72 h	┴ Cell proliferation; induced apoptosis	↑ Caspase activity; ↓ the expression of PI3K, p‐Akt and Akt; up‐regulation of miR‐200 expression	(Liu et al. 2017)
CT26	1.25, 2.5, 5, 10, 20, 40, and 80 μM, 24 and 48 h	┴ Cell growth, invasion, and proliferation; ↓ the secretion of angiogenic markers	Down‐regulation of the expression of MMP‐2 and MMP‐9; down‐regulation of VEGF, VEGFR2, and HIF‐1α	(Zhu et al. 2017)
Lung cancer				
NSCLC (A549)	5, 10, and 20 μM, 2 and 24 h	┴ TGF‐β1‐induced cell migration and invasion; ┴ TGF‐β1‐induced EMT process; ┴ MMP‐2 and MMP‐9 expression	Inactivation of Smad2/3 and NF‐κB signaling pathways	(Wang et al. 2019)
Gastric cancer				
MKN‐45 and HGC‐27	2.5, 5, 10, 20, 40, 80, and 160 μM, 24 h	↓ Cell proliferation and migration; induced apoptosis; ↑ ROS generation	Down‐regulation of the expression of MMP‐2, α‐SMA, RhoA, ROCK, N‐cadherin, and Bcl‐2; induction of Bax expression	(Wang and Zhan‐Sheng 2018)
Osteosarcoma				
MG63 and U2OS	2.5, 5, 10, 20, 40, 80, and 100 μM, 72, 48, and 24 h	↓ Cell viability; ┴ cell migration; activated both intrinsic and extrinsic apoptosis pathways	↓ Kras, RACK1, and MMP‐2 expression; ↑ of cleaved caspases and PARP; up‐regulation of Bax; down‐regulation of Bcl‐2; ↑ ROS; ┴ of STAT3/JAK2/Src	(Wang and Xue 2018)
Bladder cancer				
T24	20, 40, 80, 160, and 320 μM, 24, 48, and 72 h	↓ Cell proliferation, migration, and invasiveness; ↑ apoptosis; ↑ cell‐cycle arrest at the G_2_/M phase	Modulation of PI3K/Akt pathway; ┴ of the expression of anti‐apoptotic Bcl‐2 family proteins; modulation of caspase‐dependent pathway	(Jin et al. 2015)
Ovarian cancer				
OVCAR‐3	25, 50 and 100 μg/mL, 24, 48, and 72 h	↓ Cell proliferation, ↑ apoptosis	Up‐regulation of miR‐200, down‐regulation of MMP‐2 expression	(Gao et al. 2014)

*Note*: Various symbols (↑, ↓, and ┴) indicate an increase, a decrease, and an inhibition in the obtained variables, respectively.

Abbreviations: α‐SMA, alpha‐smooth muscle actin; Akt, protein kinase b; Bax, B‐cell lymphoma protein 2 associated X protein; Bcl‐2, B‐cell lymphoma protein 2; CRC, colorectal cancer; EMT, epithelial‐mesenchymal transition; HCC, hepatocellular carcinoma; HIF‐1α, hypoxia inducible factor α; JAK2, Janus kinase 2; miR, micro RNA; MMP, matrix metalloproteinase; MTA3, metastasis‐associated protein 3; NF‐κB, nuclear factor kappa B; NSCLC, non‐small cell lung cancer; p‐Akt, phosphorylated protein kinase b; PARP, poly‐(ADP‐ribose) polymerase; PI3K, phosphatidylinositol 3‐kinase; PTMs, post‐translational modifications; RACK1, receptor for activated C‐kinase 1; RhoA, Ras homolog gene family member A; ROCK, Rho‐associated coiled‐coil kinase; ROS, reactive oxygen species; Sirt1, Sirtuin 1; Smad2/3, Smad family member 2/3; Src, tyrosine‐protein kinase; STAT3, signal transducer and activator of transcription 3; TGF‐β1, transforming growth factor beta 1; VEGF, vascular endothelial growth factor; VEGFR2, vascular endothelial growth factor receptor 2.

**TABLE 2:** In vivo anticancer effects and underlying mechanisms of action of catalpol.

Animal model	Dose (route)	Duration	Effects on tumor	Mechanisms of action	References
Breast cancer					
BALB/c nude mice bearing MCF‐7 tumor xenografts	20 mg/kg (intraperitoneal injection)	Every other day (the total duration was not specified)	↓ Tumor volume	Induction of the mitochondria apoptosis pathway, regulation of protein PTMs	(Liu et al. 2022)
Liver cancer					
Nude mice bearing HCCLM3 tumor xenografts	10, 20, or 50 mg/kg (oral administration)	Daily for 30 days	↓ Tumor weight and volume	↑ miR‐22‐3p expression level, ↓ MTA3 mRNA expression level	(Zhao et al. 2019)
Colorectal cancer					
AOM‐induced CRC in Wistar rats	10 mg/kg (intragastric administration)	Daily for 20 weeks	┴ Formation of colon tumors, ↓ tumor malignant behavior	Regulation of the expression of Sirt1 by inducing miR‐34a	(Qiao et al. 2020)
C57BL6 mice bearing CT26 tumor xenografts	7, 14, and 28 mg/kg (intragastric administration)	Daily for 5 weeks	↓ Tumor volume; ↓ the secretion of angiogenic factors and pro‐inflammatory molecules in tumors; ↑ the secretion of anti‐angiogenic factors in tumors	Supressed the infiltration degree of lymphocytes; supressed the expression of IL‐1β, IL‐6, IL‐8, COX‐2, and iNOS; down‐regulation of VEGF, VEGFR2, and HIF‐1α	(Zhu et al. 2017)
Gastric cancer					
Athymic nude mice bearing HGC‐27 tumor xenografts	10, 20, and 40 mg/kg (the route of administration was not specified)	Daily for 21 days	↓ Tumor weight and volume, ↓ tumor cell density	Cleaved caspase 3 and PARP increased expression	(Wang and Zhan‐Sheng 2018)
Osteosarcoma					
BALB/c mice bearing MG63 tumor xenografts	12.5, 25, and 50 mg/kg (intraperitoneal injection)	Daily for 28 days	↓ Tumor size, weight, and volume, ↓ tumor cell density	Not specified	(Wang and Xue 2018)

*Note*: Various symbols (↑, ↓, and ┴) indicate an increase, a decrease, and an inhibition in the obtained variables, respectively.

Abbreviations: AOM, azoxymethane; COX‐2, cyclooxygenase 2; HIF‐1α, hypoxia inducible factor 1 α; IL, interleukin; iNOS, inducible nitric oxide synthase; miR, micro RNA; mRNA, messenger RNA; MTA3, metastasis‐associated protein 3; PARP, poly‐(ADP‐ribose) polymerase; PTMs, post‐translational modifications; Sirt1, Sirtuin 1; VEGF, vascular endothelial growth factor; VEGFR2, vascular endothelial growth factor receptor 2.

#### 3.3.1 Breast Cancer

Breast cancer is one of the most common cancers in women. Among women, breast cancer is one of the top leading causes of cancer death worldwide. Risk factors for developing breast cancer include aging, family history, increased estrogen exposure, radiation, obesity, and excessive alcohol consumption. Pathologically, breast cancer occurrence involves breast cancer gene (BRCA) BRCA1 and BRCA2 mutations. Histologically, invasive ductal and lobular carcinomas are common forms of breast cancer (Hong and Xu 2022; Menon, Alkabban, and Ferguson 2024; Smolarz et al. 2022). Catalpol at different concentrations suppressed the breast cancer MCF‐7 cell line through decreased cell viability and diminished cell proliferation. The compound also increased cancerous apoptosis, caused mitochondrial membrane potential loss, and caused enhanced ROS generation by breast cancer cells, and increased cytoplasmic cytochrome c and caspase three activity levels. These effects led to mitochondrial apoptotic pathway activation and regulation of protein post‐translational modifications (PTMs), increased microRNA‐146a (miR‐146a), and decreased matrix metalloproteinase (MMP)‐16 activity (Liu et al. 2015; Liu et al. 2022). In vivo, catalpol (20 mg/kg) suppressed tumor activity by reducing tumor volume in BALB/c nude mice bearing MCF‐7 tumor xenografts. Tumor size decreased through mitochondrial apoptosis and regulation of PTMs (Liu et al. 2022). These results highlight catalpol's potential in regulating tumor size and cancer cell survival via increased cancer cell apoptosis and decreased proliferation, diminishing the chance of breast cancer spreading through metastasis by specifically regulating MMP production by malignant cells. Other published studies on iridoid glycosides have also evaluated the potential of these species in combating MMP production by breast cancer cells. However, other MMPs, such as MMP‐2 and MMP‐9, have been assessed, and these appear to be more related to breast cancer invasiveness and metastasis, especially to lymph nodes (Li et al. 2017; Rathee et al. 2013). Besides, just one animal study regarding catalpol's anticancer activity against breast cancer has been published in the current literature. Therefore, future research must delve into the reasoning for catalpol's effects against breast cancer in additional in vivo studies to translate the results to clinical settings, potentially.

#### 3.3.2 Liver Cancer

Hepatocellular carcinoma (HCC) is the primary liver tumor most associated with chronic liver disease caused by multiple risk factors. It is a common liver cancer type in the US, and there is a growing incidence parallel to hepatitis B and C virus (HBV and HCV) infections. HCC involves somatically mutated genes such as telomerase reverse transcriptase (TERT) promoter and tumor protein p53 (p53) that initiate signaling pathways disruption, such as the cases of JAK/STAT, PI3K‐Akt–mTOR, and wingless‐related integration site (Wnt)/β‐catenin. Prognostically, classical biomarkers include Ki‐67 protein expression and the p53 gene mutation, which have been repeatedly demonstrated to correlate with poor prognosis (Asafo‐Agyei and Samant 2024; Lotfollahzadeh, Recio‐Boiles, and Babiker 2024). Hepatic metastases increase the morbidity and mortality of the patients (Griscom and Wolf 2024). Against HCC cancer cell lines (HCCLM3 and Huh7), catalpol (50 μM) significantly suppressed cancer cell proliferation and viability; therefore, it reduced cancer cell colony growth through the regulation of microRNA‐140‐5p (miR‐140‐5p) expression and the microRNA‐22‐3p (miR‐22‐3p)/metastasis‐associated 1 family member 3 (MTA3) axis signaling. In addition, HCC cell invasion and migration were also diminished, which counteracts cancer invasion and migration. Other molecular mechanisms involved decreased vimentin, a cytoskeletal protein typical of hepatic cells, expression, and increased cell cycle arrest at the G0/G1 phase (Wu, Li, et al. 2021; Zhao et al. 2019). In nude mice bearing HCCLM3 tumor xenografts, catalpol (10, 20, 50 mg/kg) significantly decreased tumor weight and volume through increased miR‐22‐3p and decreased MTA3 messenger ribonucleic acid (mRNA) expression (Zhao et al. 2019). Unlike other iridoid glycosides, catalpol modulated HCC growth and invasion via genetic manipulation. Previous studies highlighted that iridoid glycosides primarily interfere with MMP, VEGF, and cluster of differentiation expressions (Kim and Choi 2021). However, the currently available evidence on catalpol against HCC does not demonstrate that the bioactive compound modulates specific proteinases or growth factors. Instead, it proves catalpol as a genetic manipulator. Therefore, it is evident that further research must delve into the rationale behind catalpol's activities against liver cancer through a more mechanistic view, highlighting the potential alterations in growth factors, enzymes, and other proteins that catalpol can modulate within its mechanisms of action.

#### 3.3.3 Colorectal Cancer

**FIGURE 4:**
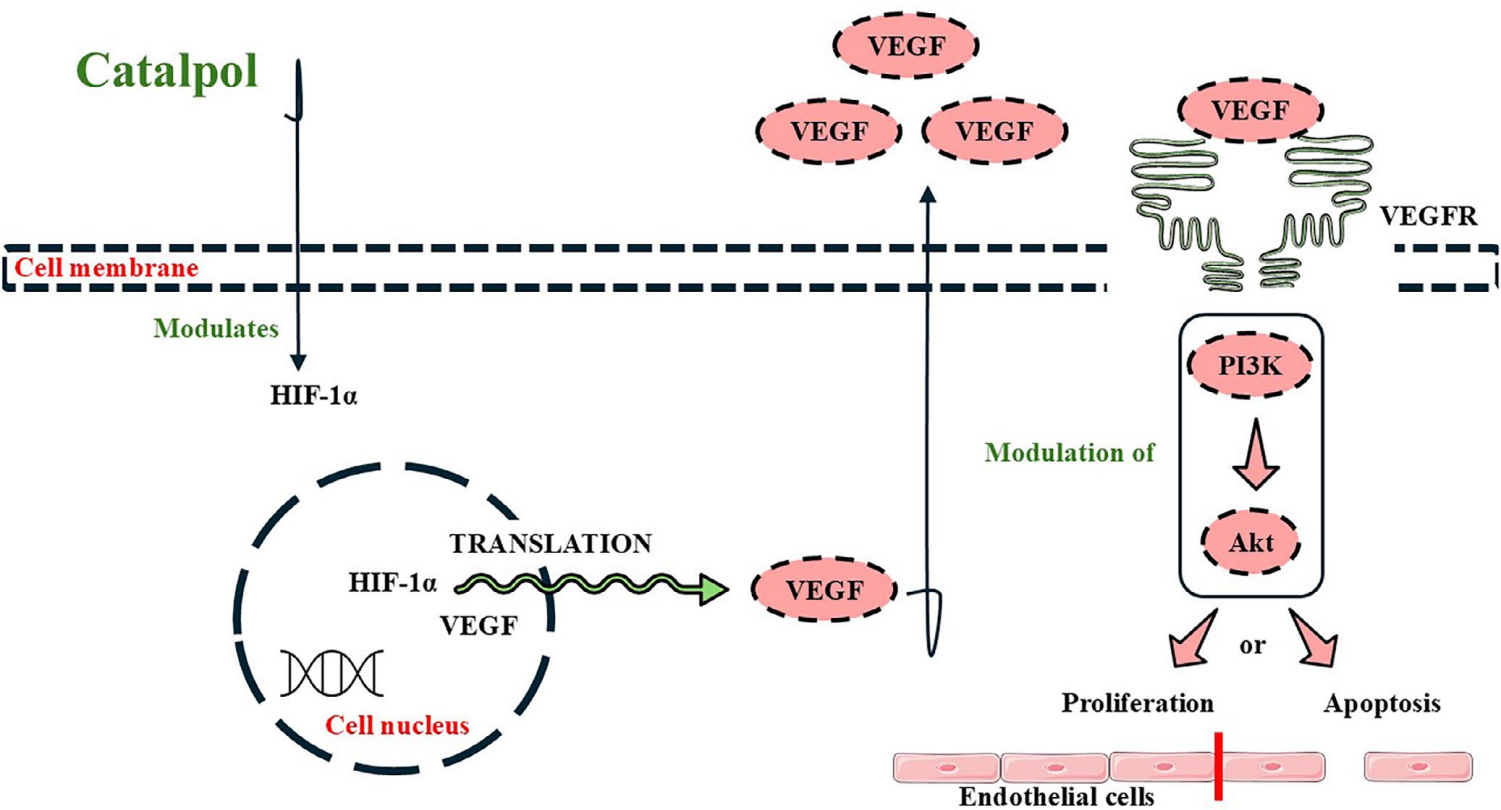
Catalpol affects angiogenesis through vascular endothelial growth factor (VEGF) production and phosphatidylinositol 3‐kinase (PI3K)/protein kinase B (Akt) signaling. HIF‐1α, hypoxia‐inducible factor 1α; VEGFR, vascular endothelial growth factor receptor. Based on Ni et al. (2024) study.

Colorectal cancer ranks as one of the most common cancers worldwide and one of the top most common cause of cancer‐related deaths in the US. Although sporadic, colorectal cancer is related to age, diet, race, and the presence of inflammatory bowel disease. However, hereditary colon malignancies can also occur, being associated with Lynch syndrome and genetic polyposis syndromes. Preventing colon cancer with a screening colonoscopy is of utmost importance to identify premalignant and early‐stage lesions. Mainly, surgery is curative. However, chemotherapy may be necessary for complete treatment. Chromosomal instability, DNA mismatch repair, and the CpG island methylator phenotype are key factors in colorectal cancer physiopathology (Lotfollahzadeh, Kashyap, et al. 2024; Menon, Recio‐Boiles, et al. 2024). Within laboratory settings, catalpol could mitigate colorectal cancer cell viability, blocking autophagy and promoting apoptosis through Sirt1 and microRNA‐34a (miR‐34a) modulation (Qiao et al. 2020). Catalpol at different concentrations also unraveled other mechanisms against colorectal cancer cell development and progression involving increased caspase activity, PI3K, phosphorylated protein kinase b (p‐Akt) and Akt downregulation, microRNA‐200 (miR‐200) increased expression, and MMP decreased production, which led to blocked cell proliferation and increased cancer cell apoptosis (Liu et al. 2017; Zhu et al. 2017). In vivo, catalpol (10 mg/kg) regulated Sirt1 expression by inducing miR‐34a, diminishing colon tumor formation, and decreasing the viability of malignant cells (Qiao et al. 2020). In C57BL6 mice bearing CT26 colon tumor xenografts, catalpol (7, 14, 28 mg/kg) decreased tumor volume and angiogenesis (Zhu et al. 2017). The results demonstrated the potential of catalpol's actions against colorectal tumorigenesis. The mechanisms by which malignant cells have decreased viability were comprehensively demonstrated by the included studies. Additionally, iridoid glycosides generally have essential anti‐inflammatory effects against colitis by inhibiting pro‐inflammatory signaling pathways like the STAT3/NF‐κB (Yuan et al. 2020). Future studies must also delve more profoundly into the possible anticancer effects of catalpol against colorectal cancer cells based on its anti‐inflammatory effects, therefore measuring pro‐inflammatory cytokines and proteins and how possible decreases in these species affect tumorigenesis and, possibly, colon tumor spread. Figure 4 demonstrates the possible effects of catalpol in endothelial cells. Against cancer, catalpol affects angiogenesis negatively, as mentioned above. However, under stress conditions like ischemic stroke, catalpol positively affects angiogenesis, increasing new blood vessel formation (Sun et al. 2023).

#### 3.3.4 Lung Cancer

Lung cancer develops in the lung parenchyma or bronchi. This type of cancer is one of the most diagnosed cancers worldwide. In the US, lung cancer represents more than 10% of all diagnoses. The risk of developing lung cancer increases with exposure to carcinogens such as asbestos, smoking, and heavy metals such as chromium, arsenic, and nickel. The pathophysiology of lung cancer is complex and not well understood. However, there are hypotheses about the continued exposure to carcinogens being the cause of lung cancer‐related genes (such as MYC, BCL2, p53, EGFR, Kras, and p16) mutations (Siddiqui et al. 2024; Thandra et al. 2021). Against lung cancer, catalpol at different concentrations was able to reduce lung malignant cell development, migration, and invasion through the blockage of transforming growth factor beta 1 (TGF‐β1) signaling and MMP expression. These effects were achieved due to inhibition of Smad family member 2/3 (Smad2/3) pathways and NF‐κB pro‐inflammatory signaling (Wang et al. 2019). These results highlight the potential of catalpol as an effective strategy for modulating critical lung cancer‐related pathways in vitro. However, other authors demonstrated iridoid glycosides also possess antiangiogenic activity in lung cancer models, an effect not shown through catalpol activity in the included study (Lou et al. 2019). Therefore, future research must elucidate the possible antiangiogenic impacts of catalpol against lung cancer to advocate in favor of the translational research involving catalpol in combination with chemotherapeutic agents against lung cancer that are primarily antiangiogenic, such as bevacizumab, ramucirumab, and nintedanib (Daum et al. 2020).

#### 3.3.5 Gastric Cancer

**FIGURE 5:**
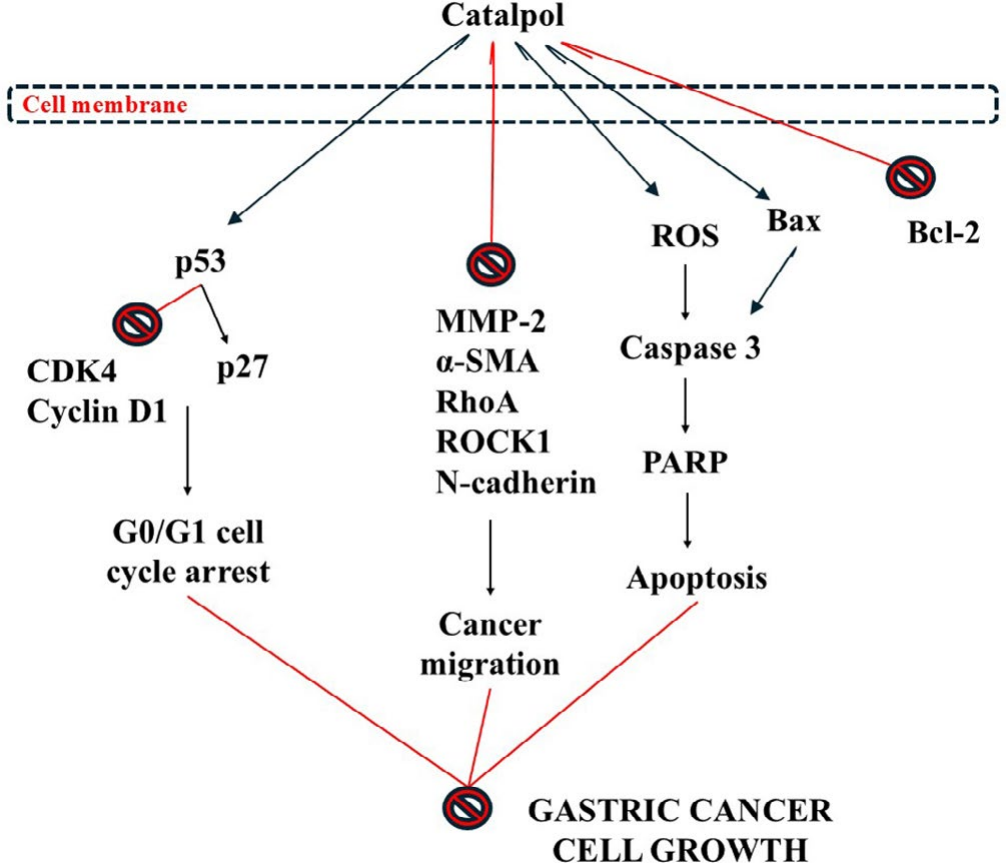
Illustration about the mechanisms by which catalpol intervenes against gastric cancer cell growth. Based on Wang and Zhan‐Sheng (2018) study. α‐SMA, alpha‐smooth muscle actin; Bax, B‐cell lymphoma protein 2 associated x protein; Bcl‐2, B‐cell lymphoma protein 2; CDK4, cyclin‐dependent kinase 4; MMP‐2, matrix metalloproteinase 2; p27, tumor protein p27; p53, tumor protein p53; PARP, poly (ADP‐ribose) polymerase; Rhoa, Ras homolog gene family member A; ROCK1, Rho‐associated coiled‐coil kinase one; ROS, reactive oxygen species. Red arrows with the block signs mean inhibition.

Gastric cancer is one of the most common in terms of diagnosis and cancer‐related mortality worldwide. The main histological subtypes of gastric adenocarcinoma are intestinal (well‐differentiated) and diffuse (undifferentiated), differing in morphological characteristics, pathogenesis, and genetic profiles. Gastric cancer pathophysiology involves AT‐rich interactive domain 1A (ARID1A) and Ras homolog gene family member A (RhoA) genetic disruptions (Mukkamalla et al. 2017; Tan and Yeoh 2015). The evidence underscores catalpol's potential to mitigate gastric cancer cell proliferation and migration through increased apoptosis and ROS generation. In MKN‐45 and HGC‐27 gastric cancer cells, catalpol at different concentrations downregulated critical molecular pathways associated with gastric cancer proliferation, such as RhoA, Rho‐associated coiled‐coil kinase (ROCK), and N‐cadherin, and suppressed MMP‐2 expression (Wang and Zhan‐Sheng 2018). In vivo, catalpol (10, 20, 40 mg/kg) significantly suppressed gastric tumor xenografts in nude mice bearing HGC‐27 gastric cancer cells through decreased tumor volume and density (Wang and Zhan‐Sheng 2018). Although the evidence underscores great catalpol's potential in modulating key pathways involved in gastric cancer progression and intervention, this potential was almost fully engaged in laboratory cellular analyses, lacking sufficient evidence to translate findings into animal studies, which present more substantial evidence for advocating for the conduct of clinical studies. Therefore, future studies must unravel the mechanisms underlying gastric cancer intervention within animal settings, highlighting the molecular pathways involved in tumor treatment outcomes. In addition, although the authors highlighted the in vitro downregulation of MMP expressions, they did not fully evaluate the invasiveness or migration of the MKN‐45 and HGC‐27 gastric cancer cells. Therefore, future studies must address catalpol's effects on invasiveness and metastasis of gastric tumor cells within in vivo studies more profoundly. Figure 5 illustrates the main mechanisms associated with gastric cancer cell growth inhibition following catalpol intervention.

#### 3.3.6 Osteosarcoma

Osteosarcoma is the primary bone malignancy derived from osteoid‐producing mesenchymal cells. In the US, hundreds of cases of osteosarcoma are diagnosed every year. Its pathophysiology needs to be fully understood. However, rapid bone growth predisposes to osteosarcoma; therefore, the cancer's bimodal age distribution (Arora and Shaikh 2024; Prater and McKeon 2024). Against osteosarcoma, one study assessed the effects of catalpol in vitro and in vivo (Wang and Xue 2018). Within laboratory settings, MG63 and U2OS osteosarcoma cell lines were treated with different concentrations of catalpol, which efficiently decreased cancer cell viability and migration and increased apoptosis. Many molecular mechanisms were up‐ or downregulated by catalpol. Indeed, the bioactive compound induced decreased Kras, receptor for activated C kinase 1 (RACK1) and MMP‐2 expression, increased cleaved caspases and poly (ADP‐ribose) polymerase (PARP) production, induced B‐cell lymphoma 2 (Bcl‐2) associated X (Bax) upregulation, promoted Bcl‐2 downregulation, and increased ROS production. Finally, the STAT3/JAK2/Src signaling pathway was blocked. In vivo, catalpol (12.5, 25, 50 mg/kg) reduced tumor size, weight, and volume, ultimately influencing tumor cell density. However, the mechanisms involved were not elucidated. Although the results are prominent, just one study assesses the impact of catalpol in osteosarcoma lineages. Therefore, future research must translate these findings into more animal studies and the first clinical trial.

#### 3.3.7 Bladder Cancer

Bladder cancer is a prevalent malignancy characterized mainly by urothelial carcinoma. In the US, bladder cancer ranks as one of the most prevalent cancers in men and in women. Early diagnosis and treatment are crucial for improving individual patient outcomes, and risk factors include smoking, chronic bladder inflammation, and continued chemical exposure to toxins like pesticides and those from painting. Bladder cancer pathogenesis is now well elucidated. However, histopathology involves non‐muscle‐invasive bladder cancer, confined to the mucosa and submucosa, and muscle‐invasive bladder cancer, penetrating the lamina propria and the superficial or deep muscle layers of the urinary bladder (Flaig et al. 2024; Leslie et al. 2024). Using the T24 bladder cancer cell line, Jin et al. (Jin et al. 2015) found that catalpol at different concentrations effectively suppressed cancer cell proliferation, migration, and invasiveness through increased apoptosis and cell‐cycle arrest at the G_2_/M phase. These effects were reached by catalpol from modulation of the PI3K/Akt pathway and controlling anti‐apoptotic proteins like Bcl‐2 and caspases. These results mechanistically demonstrate the potential effectiveness of catalpol against bladder cancer cells in vitro. However, further in vivo studies are necessary to translate findings into clinical trials. To our knowledge, the included study is the only one available on iridoid glycosides against bladder carcinogenesis. In this scenario, comparisons between other iridoid glycosides are insufficient. Comparing catalpol with other glycosides, it is worth noting that quinovic acid glycosides are efficient against bladder cancer through anti‐inflammatory pathways, modulating essential pro‐inflammatory signaling like NF‐κB (Dietrich et al. 2014; Ru et al. 2023). Therefore, future research must also evaluate catalpol's possible anti‐inflammatory effects against bladder cancer cells and the mechanisms involved in fully harnessing this iridoid glycoside's potential against this cancer type. Finally, research evaluating catalpol's effectiveness against bladder cancer invasiveness and metastasis is also of utmost importance since the spread of bladder cancer cells to profound layers of the bladder musculature or other tissues and organs is the factor that most decreases patients' outcomes (Di Bello et al. 2024; Leyderman et al. 2024; Sjödahl et al. 2024).

#### 3.3.8 Ovarian Cancer

Ovarian cancer is one of the most common gynecological cancers in postmenopausal women and usually originates from epithelial cells of the ovary. In the US, this cancer is responsible for many of female cancer deaths. The symptoms of this disease are not specific, and this contributes to late diagnosis, usually when the cancer is in an advantageous stage, which decreases the patient's survival rate. The leading risk factors are advanced age, family history, dietary fat, genetics, and hormone replacement therapy. The mechanism of this disease is not entirely understood. Still, there are many hypotheses, such as the one that suggests that ovarian cancer can be associated with non‐stop ovulation. It is known that BRCA1 and BRCA2 are genes related to ovarian cancer (Ali et al. 2023; Arora et al. 2024; Matulonis et al. 2016; Tavares et al. 2024). Catalpol has demonstrated effectiveness against ovarian cancer cells in vitro. Different concentrations (25, 50, 100 μg/mL) of the bioactive compound decreased ovarian cancer cell proliferation and increased cell apoptosis. The underlying mechanisms of action are miR‐200 upregulation and MMP‐2 downregulation (Gao et al. 2014). Future studies must address the protein and genetic signature of the ovarian malignant cells treated with catalpol to unravel more profoundly the pathways associated with decreased cell survival through increased apoptosis. Furthermore, although the included study cited MMP downregulation, it did not address the invasiveness of the treated cancer cells, remaining for future studies to address the effects of catalpol on cancer invasiveness and metastasis through in vitro and in vivo studies. In addition, the included study did not evaluate the role of catalpol in regulating epithelial‐mesenchymal transition (EMT) in ovarian cancer cells. EMT increases cell motility and invasiveness in cancer cells, and an EMT‐based genetic signature is a valuable predictor of ovarian cancer prognosis (Li et al. 2023; Pal et al. 2015; Jan and Chaudhry 2019). This would pave the way for direct cancer prevention and intervention in women presenting pre‐malignant alterations.

### 3.4 Assessing Combinatorial Anticancer Effects of Catalpol With Chemotherapeutic and Adjuvant Drugs

Conventional treatment strategies for cancer include surgery, radiotherapy, and, principally, chemotherapy (Behranvand et al. 2022; Liu et al. 2024). More recently, targeted therapies, which suppress specific molecular pathways for tumor growth and survival, and immunotherapy, which induce host immunological responses against long‐lived tumors, have also gained significant attention (Dailah et al. 2024). Although significant advancements have been made against cancer, such as stem cell therapy, radionics, chemodynamic therapy, ablation therapy, nanomedicine, and, mainly, natural antioxidants, surgical resection followed by radiotherapy with x‐rays, combined or not with chemotherapy, is still primary. Although chemotherapy has reduced the mortality and overall morbidity of cancer patients, chemotherapy agents damage healthy cells, especially those with rapidly dividing and growing cycles. In addition, growing evidence suggests that cancer cells are becoming resistant to conventional chemotherapeutic drugs due to cancerous reduced drug uptake and increased drug efflux. Dosage selection difficulty, severe side effects, lack of specificity, and rapid drug metabolism are also chemotherapy limitations (Debela et al. 2021; Kaur et al. 2023). In this scenario, catalpol has garnered significant attention due to its efficacy in combination with chemotherapeutic and adjuvant medications in several cancer lineages within preclinical models (Table 3).

**TABLE 3:** In vitro anticancer effects and underlying mechanisms of action of catalpol in combination with chemotherapeutic and adjuvant agents.

Model	Catalpol intervention	Synergism	Effects demonstrated	Mechanisms of action	References
Liver cancer					
HCC (HepG2, HUH‐7)	HepG2: 423.39 μM (IC_50_), 72 h HUH‐7: 265.59 μM (IC_50_), 72 h	HepG2: regorafenib 3.44 μM (IC_50_) + catalpol 47.48 μM (IC_50_), 72 h HUH‐7: regorafenib 5.49 μM (IC_50_) + catalpol 75.72 μM (IC_50_), 72 h	Positive synergism: ┴ cell survival and proliferation, ┴ angiogenesis	┴ PI3K/p‐Akt/mTOR/NF‐κB and VEGF/VEGFR2 signaling pathways and their downstream	(El‐Hanboshy et al. 2021)
Gastric cancer					
AGS	10, 20, 40, 80, and 160 μM, 24 h	Chloroquine 100 μM + catalpol 80 μM, 24 h	Positive synergism: ↑ apoptosis and ROS production, ┴ cell migration	Up‐regulation of Bax expression	(Sun et al. 2021)

*Note*: Various symbols (↑, ↓, and ┴) indicate an increase, a decrease, and an inhibition in the obtained variables, respectively.

Abbreviations: Bax, B‐cell lymphoma protein 2 associated X protein; HCC, hepatocellular carcinoma; mTOR, mammalian target of rapamycin; NF‐κB, nuclear factor kappa B; p‐Akt, phosphorylated protein kinase b; PI3K, phosphatidylinositol 3‐kinase; ROS, reactive oxygen species; VEGF, vascular endothelial growth factor; VEGFR2, vascular endothelial growth factor receptor 2.

#### 3.4.1 Catalpol in Combination With Regorafenib

**FIGURE 6:**
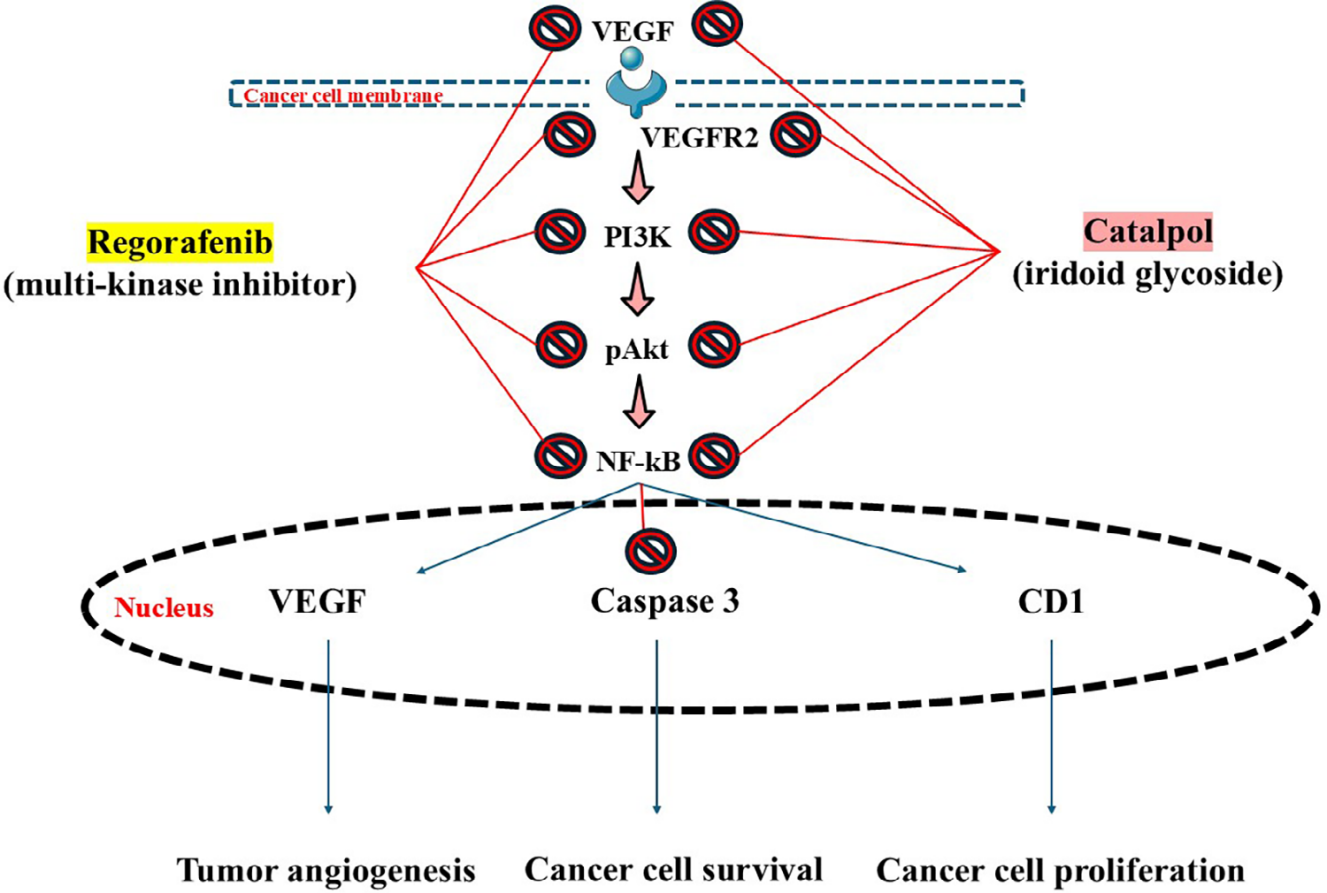
Illustration about the synergism between catalpol and regorafenib against hepatocellular carcinoma. CD1, cyclin D1; NF‐kB, nuclear factor kappa b; pAkt, phosphorylated protein kinase b; PI3K, phosphatidylinositol 3‐kinase; VEGF, vascular endothelial growth factor; VEGFR2, vascular endothelial growth factor receptor 2. Red arrows with the block signs mean inhibition. Based on El‐Hanboshy et al.'s study (El‐Hanboshy et al. 2021).

Regorafenib is the first approved drug candidate for HCC treatment in patients who have progressed through or after sorafenib chemotherapy. This chemotherapy agent is a small molecule that inhibits multiple protein kinases, such as those implicated in tumor angiogenesis and oncogenesis. Regorafenib also targets signaling pathways related to cancer apoptosis and autophagy. However, it presents several adverse effects, like hand‐foot skin reaction, fatigue, anorexia, diarrhea, hypertension, and oral mucositis (Blay et al. 2022; Carr et al. 2013; Heo and Syed 2018; Wilhelm et al. 2011). El‐Hanboshy et al. (2021) evaluated the combinational effects of catalpol with regorafenib against HCC in vitro. Synergistically, catalpol and regorafenib significantly suppressed PI3K/p‐Akt/mTOR/NF‐κB signaling in HepG2 and HUH‐7 HCC cell lines. The combinational catalpol plus regorafenib therapy also suppressed VEGF/VEGFR2 signaling pathways and their downstream effectors. Accordingly, catalpol plus regorafenib can effectively counteract cancer growth and proliferation through anti‐proliferative and anti‐angiogenic pathways, opening doors for the addition of catalpol in combinational therapies with other chemotherapeutic options that eventually do not target proliferation or angiogenesis within tumors, more specifically, HCC. Future research must endeavor to test catalpol and regorafenib in other liver tumors or even tumors of different sites, such as glioblastoma (Pinto‐Fraga et al. 2024), non‐small cell lung cancer (Aredo et al. 2024), and colorectal cancer (Sotoca Rubio et al. 2024). Figure 6 illustrates the synergism between catalpol and regorafenib against HCC based on the mechanisms of action.

#### 3.4.2 Catalpol in Combination With Chloroquine

Chloroquine (CQ) and hydroxychloroquine (HCQ) are synthetic analogs of quinine, a famous medicinal herb extract. These drugs have been widely studied against malaria. However, they have been investigated against cancer cells in various tissues and organs in recent years due to their anti‐proliferative activity as an anti‐proliferative agent. Besides, CQ and HCQ are inexpensive, rapidly orally bioavailable, and possess an exhaustive therapeutic index alongside well‐established dose safety profiles (Agalakova 2024; Cocco et al. 2022; Fong and To 2021). CQ has been synergistically tested with catalpol against AGS gastric cancer cells. The results indicated increased apoptosis through inhibition of catalpol‐induced autophagy in cultures treated with catalpol plus CQ compared to catalpol or CQ alone. Gastric cancer cells treated with the combinational treatment have also been evaluated for ROS levels, presenting increased oxidative species compared to single treatments (Sun et al. 2021). The results of this study recorded previous experiments where CQ and HCQ have substantially improved cancer treatment due to autophagy modulation (Ferreira et al. 2021). Future research endeavors must deepen the synergistic understanding of catalpol in combination with CQ or, most importantly, HCQ against cancers in which autophagy correlates with poor prognosis, such as in head and neck cancer (Debnath et al. 2023; Liu et al. 2020).

### 3.5 Assessing the Anticancer Effects of Catalpol Derivatives

Research has demonstrated that catalpol is poorly soluble within lipid environments and presents low bioavailability with a short half‐life, limiting broader clinical applications and translational research from preclinical to clinical settings. Therefore, structural modifications on catalpol have been proposed and extensively studied, including partial silylation, first silylation and then esterification of catalpol hydroxyl or total esterification, and cataloside and picroside II at the C‐6 position esterification, which are the most common modifications imposed on catalpol's original structure (Liu et al. 2021). However, other types of catalpol derivatives have been proposed for cancer targeting, especially those associated with chemotherapeutic drugs like pyrazole and imidazole, as well as other less complex ones like hydrolyzed catalpol derivatives.

#### 3.5.1 Pyrazole‐Based Catalpol Derivatives

Pyrazole derivatives are heterocyclic compounds possessing chemical structures conferring a broad spectrum of selective and potent anticancer activities. Appropriate substitutions on different positions of the pyrazole ring can significantly enhance the anticancer efficacy of another compound, as well as its tumor selectivity, by targeting tubulin, Bruton's tyrosine kinase (BTK), DNA, or cyclin‐dependent kinase (CDK) (Zhang, Ye, et al. 2023; Zhang, Wu, et al. 2023). Kong et al. designed, synthesized, and evaluated the anticancer effectiveness of pyrazole‐modified catalpol derivatives against esophageal and pancreatic cancer cell lines. The catalpol‐containing pyrazole derivative exerted strong inhibitory activity against the esophageal cancer cells (Kong, Liu, Wang, Yang, et al. 2023). This is particularly promising due to enhanced efficacy since pyrazole derivatives often target multiple proteins and signaling pathways related to cancer growth and progression. However, it is worth noting that Kong et al. did not assess the signaling pathways associated with the catalpol‐plus pyrazole‐enhanced anticancer activity. Therefore, future studies must translate these findings into in vivo studies to evaluate catalpol plus pyrazole effects in living organisms and the signaling pathways associated with these effects. Naturally, these findings must also be translated into clinical settings in well‐designed clinical trials in the future.

#### 3.5.2 Imidazole‐Based Catalpol Derivatives

Imidazole is a group of drugs that presents nitrogen‐containing heterocyclic rings. Many bioactive compounds have imidazole rings in their molecular structure. Imidazole rings often confer high polarity, hydrogen bonding, and coordination chemistry abilities, allowing imidazole derivatives to interact with various other molecular structures, including cancer‐related ones. Imidazole has been reported to interact with microtubules, histone deacetylases, PARP, p53‐Murine Double Minute 2 (MDM2) protein, and G‐quadruplexes, among other targets (Aruchamy et al. 2023; Sharma et al. 2021). Recently, Kong et al. also designed, synthesized, and evaluated the biological activities of novel catalpol derivatives containing imidazole rings. They tested the new derivatives in pancreatic cancer cells, and the results demonstrated that catalpol plus imidazole inhibited cancer growth and formation above 90% through VEGFR2 binding (Kong, Liu, Wang, Xu, et al. 2023). These results are interesting, and at this time, the authors highlighted the potential mechanism of action of the derivative catalpol plus imidazole. Targeting VEGFR2 is a practical approach to blocking pancreatic cancer because pancreatic cancer cells express VEGFR2 highly to increase cancerous growth and mobility (Doi et al. 2012; Xelwa et al. 2021). Therefore, blocking the synthesis of the growth factor would undoubtedly diminish the proliferation of the cancer and its spread throughout the body.

#### 3.5.3 Hydrolyzed Catalpol Derivatives

Previous studies have indicated that hydrolyzed glycosides have higher anticancer effectiveness than the original molecular structures. Many studies have tested hydrolyzed iridoid glycosides against malignant cells to assess their anticancer activity within laboratory settings in vitro (Ndongwe et al. 2023). Using leukemia cells, Kim et al. tested the efficacy of hydrolyzed‐catalpol (H‐catalpol) on the STAT3 signaling pathway, which is strictly correlated with leukemia cells' survival, chemoresistance, and proliferation. They found that H‐catalpol demonstrated a more significant cytotoxic effect on K562 leukemia cells than catalpol through BCR‐ABL downregulation and STAT3 inhibition through JAK2 and cellular‐Src upstream abrogation and STAT5 activation. H‐catalpol has also led to leukemia cell apoptosis via caspase‐3 activation, cyclin D downregulation, and decreased cancer cell survival through Bcl‐2, B‐cell lymphoma‐extra‐large (Bcl‐xL), and survivin inhibition (Kim et al. 2015). These results are essential due to the scarcity of other preclinical studies demonstrating the potential effects of catalpol against leukemia cells. Therefore, H‐catalpol must be tested against other hematological tumors to assess its full anticancer potential against non‐solid tumors.

## 4 Conclusions

**FIGURE 7:**
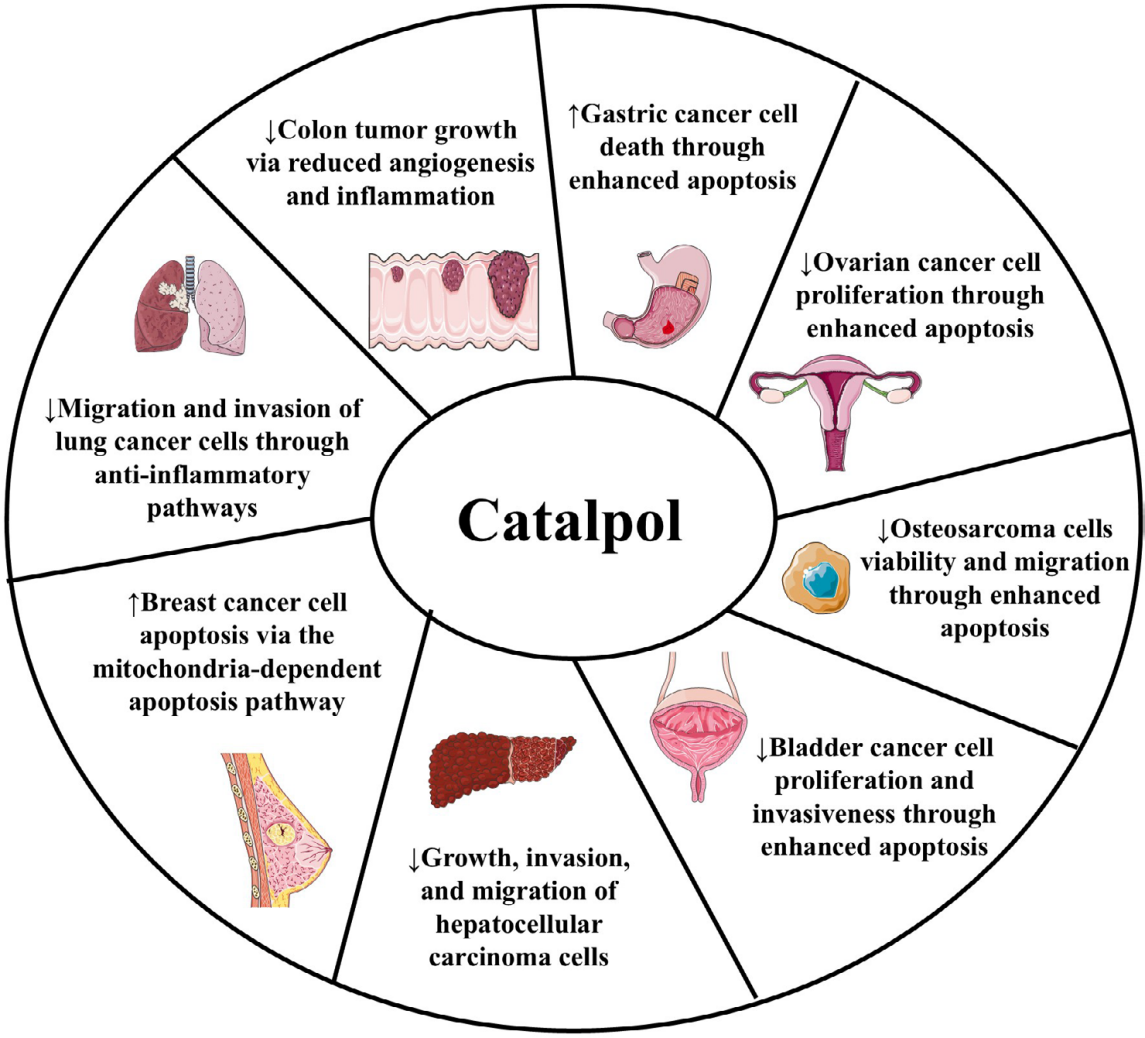
A pictorial representation of the main catalpol's mechanisms of action based on the results of the included studies.

The evidence underscores catalpol's potential in modulating several critical mechanisms associated with cancer initiation and progression. Catalpol influences several genes and proteins, impacts various signaling pathways, and modulates cancer growth and spread. Figure 7 is a pictorial representation of the main catalpol's mechanisms of action against the studied types of cancer. However, it is worth noting that the specific pathways by which catalpol influences cancer cell apoptosis are not yet fully understood. This review focuses on catalpol's anticancer mechanisms of action, highlighting its potential as an anticancer phytochemical, exerting chemopreventive and chemotherapeutic effects. However, further research is needed to address the compound's potential against malignancies fully. To address this necessity, conducting more preclinical in vivo studies involving catalpol against xenograft cancers remains essential to validate the current data. Future animal studies would not only elucidate more about catalpol's mechanisms against cancer growth, but also provide more nuanced and valuable data on how catalpol interacts with cancer in a whole organism, and its impacts against cancer spread and metastasis. Information on catalpol's absorption, distribution, metabolism, and excretion from the animals' bodies would also significantly enhance the research conducted in this bioactive compound scenario.

Also, it is essential to mention that phytochemicals with described anticancer effects may present several applications in the pharmaceutical industry. Firstly, phytochemicals with chemopreventive and chemotherapeutic actions may increase the effectiveness of synthetic or widely used chemotherapeutic drugs. Catalpol, as aforementioned in this review, has been used alongside traditional chemotherapeutic agents to modulate and enhance anticancer outcomes positively. Future research can also investigate the effects of catalpol on reducing adverse effects, toxicity, and off‐target effects of synthetic chemotherapies within animal and clinical settings. Cancer prevention is another area for the pharmaceutical industry to invest in since catalpol can be produced as a chemopreventive agent and sold mainly. Using phytochemicals as anti‐inflammatory and antioxidant agents to prevent carcinogenesis is a well‐deserved area of interest. In addition, future research must endeavor to seek possible immunomodulatory effects of catalpol against cancer. Glycosides potentiate the immune system for controlled actions. If researchers find that catalpol could reprogram neutrophil function, for example, there is another mechanism of action against metastasis (Xiong et al. 2021).

Finally, combined with nano‐delivery systems, catalpol can enhance the sensitivity of tumor cells for chemotherapy, radio‐sensitize tumors for radiotherapy, and enhance immunotherapy outcomes. Combining phytochemicals with nanomedicine is crucial because it provides valuable cooperation between the academic community and the pharmaceutical industry. Nanoformulations enhance absorption, stability, distribution, and bioavailability, enabling targeted delivery to tumors. Currently, no research has evaluated catalpol combined with nanomedicine against tumor cells. However, this is also a future research endeavor. Materials containing single‐, di‐, tri‐, and multi‐metal atoms bonded to C, N, S, P, B, and O species improve several pharmaceutical agents' biomedical applications, and this is not different from phytochemicals. Phytochemicals can also be conjugated with gold and silver to contribute to tumor imaging and diagnosis (Kim et al. 2021; Tiwari et al. 2024). The prospect of using catalpol conjugates with nanotechnology is also intriguing for the pharmaceutical industry, as it can enhance patients' quality of life in oncology through better and targeted therapies that decrease the risk of tumor relapse and metastatic disease manifestation (Koklesova et al. 2023; Sheik et al. 2023).

## Author Contributions

**Lucas Fornari Laurindo:** conceptualization, investigation, funding acquisition, writing – original draft, writing – review and editing, visualization, validation, methodology, software, formal analysis, project administration, resources, supervision, data curation. **Victória Dogani Rodrigues:** writing – review and editing, writing – original draft. **Elen Landgraf Guiguer:** writing – original draft, writing – review and editing. **Lívia Fornari Laurindo:** writing – review and editing, writing – original draft. **Debora Aparecida Pires de Campos Zuccari:** writing – original draft, writing – review and editing. **Claudia Rucco Penteado Detregiachi:** writing – original draft, writing – review and editing. **Adriano Cressoni Araújo:** writing – original draft, writing – review and editing. **Jéssica da Silva Camarinha Oliveira:** writing – original draft, writing – review and editing. **Durvanei Augusto Maria:** writing – original draft, writing – review and editing. **Jefferson Aparecido Dias:** writing – original draft, writing – review and editing. **Rose Eli Grassi Rici:** writing – original draft, writing – review and editing. **Caroline Barbalho Lamas:** writing – original draft, writing – review and editing. **Rosa Direito:** conceptualization, investigation, funding acquisition, writing – original draft, writing – review and editing, visualization, validation, methodology, software, formal analysis, project administration, resources, supervision, data curation. **Sandra Maria Barbalho:** conceptualization, investigation, funding acquisition, writing – original draft, writing – review and editing, visualization, validation, methodology, software, formal analysis, project administration, resources, supervision, data curation.

## Conflicts of Interest

The authors declare no conflicts of interest.

## Data Availability

Data sharing is not applicable to this article as no new data were created or analyzed in this study.
